# Children Facial Expression Production: Influence of Age, Gender, Emotion Subtype, Elicitation Condition and Culture

**DOI:** 10.3389/fpsyg.2018.00446

**Published:** 2018-04-04

**Authors:** Charline Grossard, Laurence Chaby, Stéphanie Hun, Hugues Pellerin, Jérémy Bourgeois, Arnaud Dapogny, Huaxiong Ding, Sylvie Serret, Pierre Foulon, Mohamed Chetouani, Liming Chen, Kevin Bailly, Ouriel Grynszpan, David Cohen

**Affiliations:** ^1^Service de Psychiatrie de l’Enfant et de l’Adolescent, GHU Pitie-Salpetriere Charles Foix, Assistance Publique – Hôpitaux de Paris, Paris, France; ^2^Institut des Systèmes Intelligents et de Robotique (ISIR), CNRS UMR 7222, Sorbonne Université, Paris, France; ^3^Institut de Psychologie, Université Paris Descartes, Sorbonne Paris Cité University, Paris, France; ^4^Cognition Behaviour Technology (CoBTeK), EA 7276, University of Nice Sophia Antipolis, Nice, France; ^5^Laboratoire d’Informatique en Image et Systèmes d’Information (LIRIS), Ecole Centrale de Lyon, CNRS, UMR 5205, 69134, Villeurbanne, France; ^6^Groupe Genious Healthcare, Montpellier, France

**Keywords:** emotion, production, facial expression, development, children

## Abstract

The production of facial expressions (FEs) is an important skill that allows children to share and adapt emotions with their relatives and peers during social interactions. These skills are impaired in children with Autism Spectrum Disorder. However, the way in which typical children develop and master their production of FEs has still not been clearly assessed. This study aimed to explore factors that could influence the production of FEs in childhood such as age, gender, emotion subtype (sadness, anger, joy, and neutral), elicitation task (on request, imitation), area of recruitment (French Riviera and Parisian) and emotion multimodality. A total of one hundred fifty-seven children aged 6–11 years were enrolled in Nice and Paris, France. We asked them to produce FEs in two different tasks: imitation with an avatar model and production on request without a model. Results from a multivariate analysis revealed that: (1) children performed better with age. (2) Positive emotions were easier to produce than negative emotions. (3) Children produced better FE on request (as opposed to imitation); and (4) Riviera children performed better than Parisian children suggesting regional influences on emotion production. We conclude that facial emotion production is a complex developmental process influenced by several factors that needs to be acknowledged in future research.

## Introduction

From an early age and throughout one’s lifespan, emotional skills are essential to communicate our emotions to others and to modulate and adapt our behavior according to both our internal feelings and the reaction of others (Saarni, 1999; Halberstadt et al., 2001). The ability to understand what we feel, to deal with our own emotion and that of others, and to show emotional empathy are factors of integration in the society at all ages of life. Although our experience of the world is multimodal (we see objects, hear sounds, feel texture, smell odors, and taste flavors), visual signals and languages are key social signals in humans (Adolphs, 2003). Among visual signals, facial expressions (FE) are crucial components of emotional signals. They allow people to understand and express not only emotions (Izard, 1971; Izard, 2001) but also social motivation (Fridlund, 1997).

Facial expressions recognition has been investigated in numerous studies, showing that many variables can influence the interpretation of FEs: (i) FE recognition increases during childhood with the age of the perceiver (Herba et al., 2006; Lawrence et al., 2015) and declines for older adults compared to young adults (see Ruffman et al., 2008). (ii) Modality influences emotion recognition, and multimodal supports are easier to recognize than unimodal supports (Castellano et al., 2008; Luherne-du Boullay et al., 2014). (iii) The condition of presentation from static or dynamic support is also important (Biele and Grabowska, 2006; Trautmann et al., 2009). (iv) FE are more easily recognized when the producer is younger rather than older (Fölster et al., 2014). (v) Girls are generally more efficient in identifying emotion (Hall et al., 2000; Lawrence et al., 2015) but not all studies support this conclusion (Herba et al., 2006). Some differences in methodology could explain these differences, as the choice of the intensity of the expressions (Hoffmann et al., 2010). (vi) Emotion recognition is higher when emotions were both recognized and expressed by members of the same regional group (Elfenbein and Ambady, 2002). Moreover, majority group members are poorer at judging minority members than the reverse. (vii) The context in which FE is produced can also contribute to emotion recognition (Wallbott, 1988; Mobbs et al., 2006). (viii) The different emotional FEs themselves are not equally identified: joy appears to be one of the easiest FE to be recognized (Lawrence et al., 2015).

Facial expressions production has received less attention than FE recognition in the literature. There are mainly three methods to evaluate FE production. The first is the measure approach which describes and measures objectively observable and measurable changes of facial components. The most widely used method is the Facial Action Coding System (FACS, Ekman et al., 2002) which requires a trained expert to rate. The second and the most commonly used in the establishment of a dataset is the judgment approach introduced by Darwin (1872) which is based on the fact that everyone can relate a FE to an emotion. This method consists of presenting FE to a sample of judges, and the accuracy of the FE is inferred thanks to their rating. In most previous studies (Egger et al., 2011; Dalrymple et al., 2013), researchers recorded individuals when they produced a FE. Then, blind annotators had to rate the video in two steps: first, they had to first identify which emotion was produced and then had to rate its intensity. Few studies try to rate the quality of the emotion, and the way to do it is not consensual. In studies of children, Egger et al. (2011) asked the judges how well the emotion was portrayed. Mazurski and Bond (1993) looked at the certainty of the judge that the emotion he recognized was the good one. In studies of adults, such as the GEMEP (Bänziger et al., 2012), the judges had to rate the authenticity and the plausibility of the FE. The third method to assess FE is based on algorithmic automatic assessments trained on large datasets that provide a normed FE material (Zeng et al., 2009). However, this method requires the algorithm to be previously trained on a dataset already rated by human judges.

To date, most of the datasets describing a large dataset of FE concern adult FE. In the most recent studies, the datasets propose both static and dynamic sequences with different face orientations (Pantic et al., 2005), multimodal production (Bänziger et al., 2012) as well as played (e.g., professional actors) or natural facial productions (Zhang et al., 2014). But very few datasets concern FE of children (see **Table 1**). Moreover, most of them include only static 2D supports (mainly photographs). The Facewarehouse dataset is the only one made of 3D video recordings of FE, but it does not include just children nor does it indicate how many children are involved (Cao et al., 2014).

**Table 1:** Databases that include children facial expressions.

Databases	Population	Emotions	Support
NIMH-ChEFS (Egger et al., 2011)	39 girls and 20 boys from 10 to 17 years old	Fear, anger, happiness, sadness, and neutral	482 photographs
Dartmouth database (Dalrymple et al., 2013)	40 caucasian girls and 40 caucasian boys from 6 to 16 years old	Neutral, satisfaction, happiness, sadness, anger, fear, surprise, and disgust	Photographs
Facewarehouse (Cao et al., 2014)	150 people from 7 to 80 years old (proportion of children unknown)	Mouth stretch, smile, brow lower, brow raiser, anger, jaw left, jaw right, jaw forward, mouth left, mouth right, dimpler, chin raiser, lip puckerer, lip funneler, sadness, lip roll, grin, cheek blowing, and eyes closed	3D Vidéos
Japanese database (Komatsu and Hakoda, 2012)	53 boys et 54 girls from 11 to 13 years old	Neutral, happiness, surprise, anger, and sadness	535 photographs
Slides depicting facial expression of affect (Mazurski and Bond, 1993)	3 boys (9 to 11 years old) et 3 girls (8 to 12 years old)	Anger, disgust, fear, happiness, surprise, and neutral	Photographs
CAFE (LoBue and Thrasher, 2014)	90 girls and 64 boys from racially and ethnically diverse group between 2 and 8 years old	Anger, fear, sadness, happiness, neutral, surprise, and disgust	1192 photographs

Most studies regarding FE production were conducted in adulthood. Ekman et al. (1987) defined six emotions as universal (sadness, happiness, anger, surprise, fear, disgust, also combined with contempt), common among all humans, independently of culture or origin. Nowadays, this theory is questioned. If it is generally accepted that these six emotions are innate for a part, new studies show that culture can modulate FE production (Elfenbein et al., 2007). Moreover, other factors influence FE production. Women are described as more expressive than men (Brody and Hall, 2000). They tend to produce more positive emotions while males express more anger. FE production is also influenced by the context around the producer. FE of a participant is better recognized if he produces it in presence of a friend than in presence of a stranger (Wagner and Smith, 1991). People produce more easily FE of happiness in pleasant situations with people but tend to hide negative FE in unpleasant situations with people around them (Lee and Wagner, 2002).

In terms of development, it appears that most of the facial components of human expression can be observed shortly after birth like expression of enjoyment and interest that are present from the opening days of life (Sullivan and Lewis, 2003). Researcher first thought that infant FEs corresponded to adults FEs (see Differential emotion theory in Izard and Malatesta, 1987), but it’s now known that FEs in infancy are not present like their adult-counterparts (Oster, 2005). The first reason is that emotion in infancy cannot be compared to emotion in adulthood. Sroufe (1996) described precursor emotions in infancy which do not involve some degree of cognitive evaluation like for emotions in adults. He described wariness and frustration that are similarly manifested in crying and distress. This observation concurs with the study of Camras et al. (2007) that do not find different FEs for fear and anger at 11 months. Another reason of differences between adult and infant FEs could be linked to the motor structure of infant face. Camras et al. (1996) noted that infants may produce FEs in a non-related situation because of an enlarged recruitment among facial muscles during movement. For example, infants of 5 and 7 months raise their brows as they open their mouth, producing an expression of surprise.

Holodynski and Friedlmeier (2006) proposed that infants learned adult-like expressions thanks to a sociocultural based internalization model; caregivers reproduced infant expressions in a selective and exaggerated form, allowing children to learn the concordance between their emotion and a given FE.

However, the apparition of adult-like expressions is not well known (Oster, 2005). Bennett et al. (2005) showed that the organization of facial expressivity increases during infancy. 12-month infant showed more specific expression to a situation than 4-month infants. In response to tickle, the number of infants exhibiting joy expression increased and the number exhibiting other expressions (like surprise or interest) decreased. It seems that children continue to learn how to produce FE even in late childhood. Ekman et al. (1980) showed that the ability to produce FE improves between 5 and 13 years. However, they do not perfectly produce all FE. In the same way, Gosselin et al. (2011) showed that children between 5 and 9 years old activated unexpected action components when they were asking to produce sadness and joy.

The subtype of emotion can also influence productions of children. Brun (2001) studied the FE in children between 3 and 6 years old. The children had to evoke the FE from a sound link to an emotion. The production of FE depends on age and the targeted emotion: joy is already well produced at 3 years old while anger, sadness and surprise are still not mastered at 6 years old. Field and Walden (1982) also found that positive emotions are easier to produce than negative emotions. However, LoBue and Thrasher (2014) asked children to imitate FE of an adult and found no effects of age or emotion subtype on the production of FE for children between 2 and 8 years old.

Most studies assessed the effect of gender on emotion production with girls that produce more positive FE and boys more negative FE. During adolescence, gender differences have been reported with (i) judges rating girls’ positive expressions stronger than boys’ productions, and boys’ expressions of anger, sadness, and surprise stronger than girls’ expressions (Komatsu and Hakoda, 2012); and (ii) with girls smiling more often than boys (LaFrance et al., 2003). However, LoBue and Thrasher (2014) found no effect of gender on FR production for children between 2 and 8 years old. Effectively, the effect of gender seems to be modulating by other factors. Chaplin and Aldao’s (2013) meta-analytic review confirmed the interaction between gender, age and type of emotion during FE. They found no gender difference in infancy and preschoolers. However, they found that children and adolescent girls express more positive emotion than boys. Conversely, a small effect of gender appears in infancy, preschoolers and childhood but disappears in adolescence for the production of internalizing emotions (such as sadness or sympathy) with more accuracy for girls. For externalizing emotions (like anger), they found no difference in infancy. But boys were better than girls in production during childhood. Unexpectedly, the differences reverse in adolescence with better productions of externalizing emotions for girls than for boys.

As in adults, ethnicity and culture seems to influence FE production. Comparing four groups of 3 year old girls (European–American, Chinese girls adopted in a European–American family, non-adopted Chinese–American girls and Chinese girls living in mainland China), Camras et al. (2006) found that European–American girls were more expressive than Chinese–American girls and mainland Chinese girls. Adopted Chinese girls generally fell between the European–American group and the 2 other Chinese groups. They differed significantly from the 2 other Chinese groups for disgust. The influence of ethnicity is also shown by Louie et al. (2013). They found that preschooler of Asian American parents and from Korean parents tend to be less expressive than preschoolers from European American family for sadness and exuberance. These findings showed that ethnicity can influence the production of emotion but also that culturally based family environment modulates the effect of ethnicity. Moreover, this effect seems to appear in the 1st year of life (Camras et al., 2007; Muzard et al., 2017).

So far, very few studies have proposed to study spontaneous production of FE (e.g., Sato and Yoshikawa, 2007). Most of the time, the targeted population produces FE on request (e.g., Egger et al., 2011; Dalrymple et al., 2013). However, FE can be produced while imitating a model (e.g., a picture, a drawing, a video of a virtual agent or another human like in LoBue and Thrasher, 2014). In the current paper, we will call this type of tasks “imitation” as opposed to FE production “on request” (e.g., an oral or writing order, or pictures or oral contexts without model).

Also, few research targeted FE in children. They supposed that many variables could influence children’s productions as gender, culture, emotion subtype, but data are missing to understand the effects of these variables through age. Open questions remain regarding typical child performances in producing FE between 6 and 11 years old. Moreover, the influence of the type of tasks and the modality in which they are presented are not well documented. The first aim of our work is to explore the quality of the FEs of children between 6 and 11 years old. We tested the capacities of typical children to produce FE on demand and the several moderating variables such as age, gender, type of emotion, condition of production (visual vs. bimodal), context of elicitation (imitation vs. acting on request) and region (Parisian vs. French Riviera) that could influence their productions. We hypothesized performance to increase with age, girls to perform better than boys, positive emotions to be easier to produce than negative emotions, bimodal presentation to make FE easier to produce than visual unimodal presentation, imitation to make FE easier to produce than acting on request, and Mediterranean children to perform better than Parisian children.

The current work enters into the larger project, JEMImE, intended to improve FE of children with ASD. Children with ASD have difficulties to identify and produce adapted FE (Uljarevic and Hamilton, 2013; Gordon et al., 2014). The JEMImE project aims to create a serious game to stimulate children with ASD to produce adapted FE in context. To reach this goal the game inspired by JeStimule, that aims to train emotion recognition in children with ASD (Serret et al., 2014), will automatically score online children’s FE production to help the child (or the therapist) to monitor his production. In order to provide this feedback an algorithm that is able to recognize in real time the production of the player will be integrated into the game. To deal with the lack of extended datasets with children producing FE, we had to record a large dataset. The second aim of our work is so to capture and rate a large dataset of children’s FEs in order to train the algorithm (Grossard et al., 2017).

## Materials and Methods

### Participants

Children were recruited in two French public schools, one in Paris, one in Nice, from January 2015 to January 2016. The two schools were not located in areas known to be recruiting a high rate of children with socio-economic or developmental risk^1^. We only recruited native French children. In total, 157 children aged between 6 and 11 years old (boys, *N* = 52%; girls *N* = 48%) were enrolled in the study. Origins were varied but we included more Caucasian children (77.1%), and fewer African children (8.3%), Asian children (7%) and Maghreb children (7%). The percentage of Caucasian children was higher in Nice (89.7%) than in Paris (58.7%). Before inclusion, written consents were obtained after proper information from school directors, parents and children. Each child was met alone during approximately 40 min to complete the protocol. The study was approved by the ethical committee of Nice University (*Comité de Protection des Personnes Sud Méditerranée*) under the number 15-HPNCL-02.

### Tasks

Two tasks (demands of FE production on request and by imitation) were proposed. The two tasks were chosen in order to collect productions with and without a model (here an avatar) and thus to compare facial production in the two different tasks. Children had to produce four FEs: joy, anger, sadness, and neutral.

In the imitation task, the child must imitate the facial productions (visual modality) and the facial and vocal productions (audiovisual modality) of an avatar presented on his screen in short videos of 3–4 s. Two avatars (1 boy/1 girl) were created for this tool in order to counteract a possible gender effect of the model on FE recognition. These avatars were first tested with 20 adults who had to recognize the emotion produced and reach a recognition rate above 80%. Each of the avatars produced the four emotions. The avatars and the FEs were presented in a random order. The audiovisual condition combines FEs with emotional noises (such as crying for sadness, rage for anger or pleasure for joy, a/a/ held for neutral emotion). These sounds were extracted from an audio dataset validated in adults (Belin et al., 2008).

In the production on request, the child had to produce a FE (visual modality) or a facial and vocal expression (audiovisual modality) on request. The name of the emotion was displayed on the computer screen and read by the clinician. The order of presentation of emotions within this task was also random.

### Design and Recording

**FIGURE 1:**
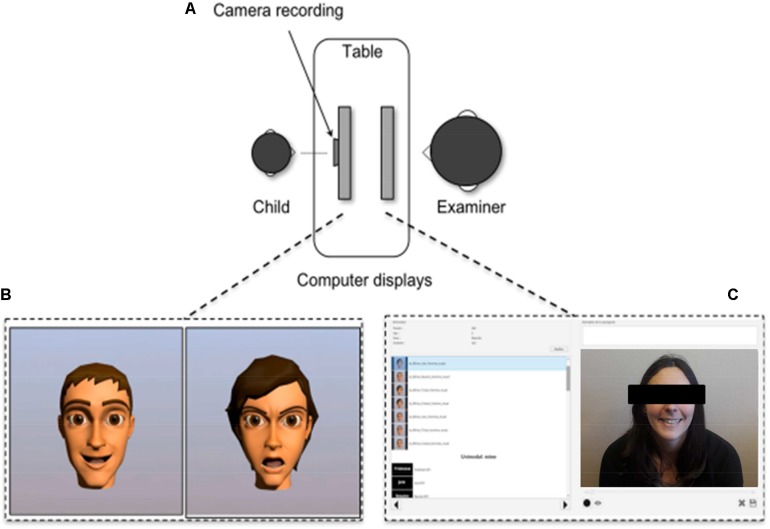
Design and recording of the FE tasks. **(A)** Installation during the recording; **(B)** children screen showing two avatars showing two different FE; **(C)** Examiner control screen. Written informed consent was obtained from the participant for the publication of this image.

Each child produced each emotion twice on request and four times in imitation (**Figure 1**). We doubled the imitation condition in order to have enough trials with avatars of both genders. The two tasks were first proposed in visual condition alone, then in audiovisual condition (facial and vocal). For each modality, they were proposed in a random order to avoid a learning effect (**Figures 1A,B**) and the modality presentation (visual modality vs. audiovisual modality) was counterbalanced. Each of this order was balanced according to gender and age (**Table 2**).

**Table 2:** Repartition of children according to age, gender, site and order of presentation.

Age	Sex	6–7 years			7–8 years			8–9 years			9–10 years			10–11 years			Total		
Site		Nice	Paris	All	Nice	Paris	All	Nice	Paris	All	Nice	Paris	All	Nice	Paris	All	Nice	Paris	All
	Girls	5	1	6	7	1	8	3	1	4	2	1	3	2	1	3	19	5	24
Order 1	Boys	6	1	7	7	3	10	3	2	5	2	2	4	2	2	4	20	10	30
	Girls	3	1	4	1	2	3	2	1	3	3	2	5	2	1	3	11	7	18
Order 2	Boys	1	2	3	1	3	4	2	1	3	2	3	5	0	1	1	6	10	16
	Girls	3	0	3	0	1	1	2	2	4	3	1	4	3	1	4	11	5	16
Order 3	Boys	1	1	2	1	4	5	2	1	3	2	2	4	2	2	4	8	10	18
	Girls	3	1	4	1	1	2	2	2	4	2	2	4	3	1	4	11	7	18
Order 4	Boys	1	2	3	1	3	4	2	2	4	2	1	3	2	1	3	8	9	17
	Girls	14	3	17	9	5	14	9	6	15	10	6	16	10	4	14	52	24	76
	Boys	9	6	15	10	13	23	9	6	15	8	8	16	6	6	12	42	39	81
Total	Children	23	9	32	19	18	37	18	12	30	18	14	32	16	10	26	94	63	157

Each child was video recorded for 2–3 s using a 2D/3D video camera. Each video contained one FE. During the recording children had their own screen and the examiner had another. The examiner was seated in front of them in order to avoid that children turn their head out of the screen (**Figure 1**).

### Imitation Task Instruction

The following instructions were given:

– [visual modality]: “You will see an animated face on the screen. It will produce an emotion with his face, like joy for example. You’ll have to do the same thing with your face.”
– [audiovisual modality]: “You will see an animated face on the screen. It will produce an emotion with his face and his voice, like joy for example. You’ll have to do the same with thing with your face and your voice.” We collected 16 videos per child.

### On Request Task Instruction

The following instructions were given: “I will tell you a word which expresses an emotion when we feel something:

– [visual modality]: Could you show with your face what you do when you feel sadness/joy/anger/nothing?”
– “[audiovisual modality]: Could you show with your face and your voice what you do when you feel sadness/joy/anger/nothing?” We collected eight videos per child.

### Coding

To analyze the productions of the children, all the videos recorded needed to be annotated. For our purpose we chose to keep a more naturalistic way of rating emotion. Indeed, the serious game JEMImE is aimed at teaching children with ASD how to produce adapted FE in the most natural way. We had to look for how to judge the quality of an FE, which is not consensual in the literature. To construct our coding tools, we decided to consider the quality of an FE like a combination of recognizing and credibility. By postulating that if the emotion cannot be recognized it cannot be credible, it is possible to create a continuum between recognition and credibility. Indeed, we decided to create a scale from 0 to 10 where 0 corresponds to the absence of the expression, 5 to the recognition of the emotion but it does not seem credible and 10 to an emotion that is recognized and credible. Like the other tools, this scale allows to judge the presence of the emotion (0 = no recognition vs. 5 = recognition) and its quality (5 = recognition without credibility vs. 10 = recognizing and credible emotion). For each video, the judges had to complete four scales (one for each emotion: happiness, sadness, anger, and neutral). This method allows the judge to annotate one to four emotions for an expression. Indeed, a perfect production of happiness would be rated 10 in the scale for happiness and 0 on the three other scales. But for a less-specific expression (such as when children laugh while trying to produce anger), the judges would annotate multiple emotions for a unique expression (like anger 5 and joy 5). In terms of algorithmic purposes this may be of interest.

We asked three judges to annotate all the videos. The judges were French Caucasian adults (2 women and 1 man) aged 25, 34, and 40 years. They were all cognitive or developmental psychologists. The videos were blindly rated thanks to a special tool created for that purpose. In order to assess the reliability of the tool and the rating method, we asked two judges to independently annotate 10 children (240 videos in total). Children were chosen according to age, gender and presentation order of the tasks. Inter-agreement was assessed using intraclass correlation coefficients. We found excellent rates between the two judges for Happiness (ICC = 0.93), Anger (ICC = 0.92), Sadness (ICC = 0.93), and Neutral (ICC = 0.93).

### Statistical Analysis

The data of the present study were analyzed using the statistical program R, version 3.3.1 (R Foundation for Statistical Computing), with two-tailed tests (see Supplementary Data Sheet S1). The variable to be explained was the FE rating score of the expected emotion. The distribution was not normal and followed mainly a bimodal distribution with two peaks: the first peak was close to zero and the second close to 10 and only 23% of all coding scores were between 3 and 7. All attempts to transform FE rating score into a variable reaching normal distribution failed. Therefore, we transformed the FE rating score into a binary variable: failure for all scores < 5 and success for all score ≥ 5. We first explored whether each variable [gender, age, and emotion (joy, neutral, anger, or sadness), presentation order, sex of the avatar, presentation modality (visual vs. bimodal), elicitation task (imitation vs. on request), and sites (Paris vs. Nice)] was associated or not with FE rating score with bivariate analysis. Then we used a Generalized Linear Mixed Model (GLMM; lme4 and lmerTest packages) to explore the data. Given the number of observations, all variables were included in the multivariate model with the exception of the support, which was strongly dependant on the elicitation task. A binomial family was specified in the GLMM model to estimate the log-odds ratio for the corresponding factors in the model. Factors included could be gender (boy vs. girl), age, emotion (joy, neutral, anger, or sadness), presentation order, sex of the avatar, presentation modality (visual vs. bimodal), elicitation task (imitation vs. on request), and sites (Paris vs. Nice).

Finally, we also tested interactions between age, gender, and emotion as exploratory analysis given the previous results in the literature (see section “Introduction”).

## Results

### Emotion Production According to Age, Gender, and Tasks

**FIGURE 2:**
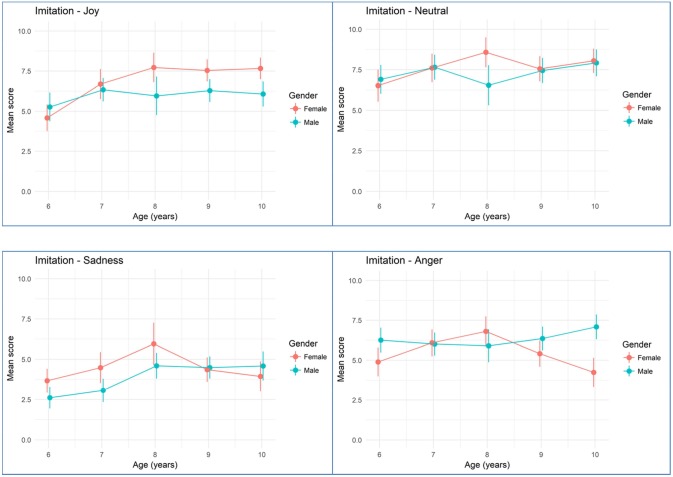
Mean emotion production scoring during the imitation task according to age and gender. Error bars are 95% bootstrapped confidence intervals.

**FIGURE 3:**
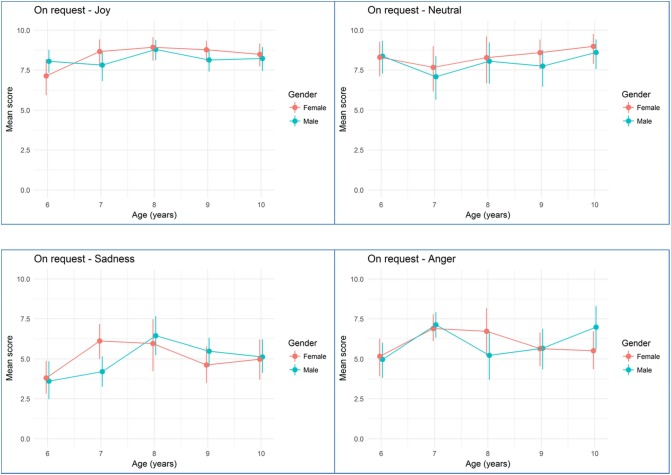
Mean emotion production scoring during the on request task according to age and gender. Error bars are 95% bootstrapped confidence intervals.

**Figures 2**, **3** show mean rating scores of children emotion production according to age and gender for imitation (**Figure 2**) and on request tasks (**Figure 3**). Bivariate analyses showed that there was a significant effect for age with higher scores for older children (β = 0.131, standard error = 0.04, *p* < 0.001) but no effect of gender (β = 0.066, standard error = 0.120, *p* = 0.584). There was no significant effect for the order of presentation (β = -0.005, standard error = 0.053, *p* = 0.918), for the visual modality vs. the audiovisual modality (β = 0.098, standard error = 0.076, *p* = 0.198). However, we found several effects for elicitation task, with the on request elicitation showing higher rating scores than imitation (β = 0.53, standard error = 0.083, *p* < 0.001), for emotion with the best scores obtained with neutral, then happiness, then anger and finally sadness (neutral vs. sadness: β = 1.68, standard error = 0.111, *p* < 0.001; happiness vs. sadness: β = 1.43, standard error = 0.107, *p* < 0.001; anger vs. sadness: β = -0.909, standard error = 0.1, *p* < 0.001), and for sites with children from Nice showing higher scores than Parisian children (β = 0.28, standard error = 0.12, *p* = 0.022).

### Multivariate Analysis

We kept in the GLMM the following explanatory variables: age, gender (boys vs. girls), order, modality (visual vs. audiovisual), emotion (joy, neutral, anger, or sadness), elicitation task (imitation vs. on request), and sites (Paris vs. Nice) (**Table 3**). The model formulation became: number of successes for the expected emotion ∼ Age + Gender + Order + Modality + Elicitation task + Emotion + Sites + (1/child name). Emotion production significantly increased with age, was easier during the on request elicitation task (as opposed to the imitation elicitation task), was easier for positive emotion than negative emotions and within negative emotion easier for anger than sadness, and finally was easier for children from Nice than from Paris. Since the most difficult emotion to produce appeared to be sadness, we calculated the model adjusted odd ratios with sadness as the referential emotion. Emotion rating score significantly increased with a factor 1.14 when the child’s age increases by 1 year. During on request elicitation task, emotion rating score significantly increased by a factor 1.71 compared to the imitation task. Emotion rating score significantly increased by a factor 5.39 for neutral, by a factor 4.20 for happiness, and by a factor 2.48 for anger compared to sadness. Finally, emotion rating score significantly increased by a factor 1.33 for Mediterranean participants compared to Parisian ones.

**Table 3:** Emotion production as a function of age, gender, order, modality, elicitation task, emotion and sites: results from the GLMM model.

Variable	β estimate	Standard error	*p*
Age	0.131	0.04	0.001
Gender (boys vs. girls)	0.066	0.120	0.584
Order	-0.005	0.053	0.918
Modality (visual vs. audiovisual)	0.098	0.076	0.198
Elicitation task (on request vs. imitation)	0.536	0.083	<0.001
Emotion (happiness vs. sadness)	1.434	0.107	<0.001
Emotion (neutral vs. sadness)	1.684	0.111	<0.001
Emotion (anger vs. sadness)	0.909	0.100	<0.001
Site (Nice vs. Paris)	0.283	0.124	0.022

Finally, we tested interaction between age, gender, and emotion. Two way interactions were estimated from two models run separately. The model formulations became: number of successes for the expected emotion ∼ Elicitation task + Order + Modality + Age + Emotion^∗^Gender + Sites + (1/child name); and number of successes for the expected emotion ∼ Elicitation task + Order + Modality + Age^∗^Gender + Emotion + Sites + (1/child name). Three way interactions were estimated from another model run separately. The model formulation became: number of successes for the expected emotion ∼ Elicitation task + Order + Modality + Age^∗^Emotion^∗^Gender + Sites + (1/child name). Two and three way interactions are summarized in **Table 4** with sadness as the referential emotion. We did not find a significant interaction between age and gender. FE expression did not increase faster with age in boys or girls (adjusted odd ratio = 1.03). We found a significant interaction between anger (as opposed to sadness) and gender. Compared to the productions of anger for girls, emotion rating increased by a factor 1.68 for boys (adjusted odd ratio). Finally, we found two significant interactions between age and gender and emotion subtypes. For the production of joy (as opposed to sadness), we found a negative interaction with age and gender. The production decreased by a factor 0.56 for boys and age (adjusted odd ratio) meaning that age increases girls ability to produce joy compared to boys by a factor 1.79 (1/0.56). Note that it doesn’t mean that girls produce joy better than boys. A similar interaction was found between the production of neutral FE (as opposed to sadness) and age and gender. The production decreased by a factor 0.72 for boys and age.

**Table 4:** Interaction model between age, gender and emotion with sadness as the referential emotion modality.

Variable	β estimate	Standard error	*p*
**Model with 2-way interaction (age^∗^gender)**			
Gender (boys vs. girls) ^∗^ Age	0.028	0.08	0.728
**Model with 2-way interaction (emotion^∗^gender)**			
Emotion (joy) ^∗^ Gender (boys vs. girls)	-0.141	0.212	0.505
Emotion (neutral) ^∗^ Gender (boys vs. girls)	-0.013	0.221	0.954
Emotion (anger) ^∗^ Gender (boys vs. girls)	0.516	0.199	0.010
**Model with 3-way interaction (age^∗^emotion^∗^gender)**			
Emotion (joy) ^∗^ Gender (boys vs. girls) ^∗^ Age	-0.584	0.149	<0.001
Emotion (neutral) ^∗^ Gender (boys vs. girls)^∗^ Age	-0.325	0.151	0.031
Emotion (anger) ^∗^ Gender (boys vs. girls) ^∗^ Age	-0.158	0.137	0.247

## Discussion

The aim of this study was to evaluate the quality of the production of FE by children on demand, the development of this ability and some factors that could influence it. Recognition of FE is well documented and the six emotions described by Ekman et al. (2013) are well recognized between 6 and 11 years. However, few studies have analyzed the production of FE in childhood. This lack of data can be explained by the difficulty to implement a protocol adapted to children, to recruit a large population, to collect the data (especially video recordings which need specific material and installation) and to rate them appropriately. Thanks to our protocol, we recorded 3875 short videos of 157 children between 6 and 11 years of age producing FEs of joy, anger, sadness and neutral expressions and rated them in terms of recognition quality and credibility. This dataset will be used to train an algorithm to recognize in real time the FE of children when playing with the serious game JEMImE computed to train FE and recognition in social contexts (Grossard et al., 2017). It will allow them to adjust their productions thanks to real time feedbacks.

As expected, the accuracy of FE emotional production increased with age. Whatever the other moderators, the FEs are best produced in older children. But it is important to note that children did not produce FE perfectly well, even for the oldest children (e.g., mean score at 10 years old is 6.5/10).

Other significant moderators of the quality of FE include the targeted emotion. For example, the score for the production of anger oscillate between 5 and 7.5 (for a maximum of 10), whatever the task. We expected that positive emotions would be easier to produce than negative emotions. Effectively, joy is produced with more accuracy than anger or sadness. Neutral emotion remains the state the most easily produced. However, in the on request task, joy is produced as well as neutral, even by young children (**Figure 3**). These findings concur with the observation of Brun (2001) demonstrating that joy is the emotion the most quickly mastered by children. Sadness is the emotion produced with less accuracy. These differences between positive and negative emotions may also come from the context of the signing. In adulthood, Lee and Wagner (2002) found that participants tend to hide their negative emotion when there are people around. In our protocol, some children tend to laugh when they had to produce negative emotion, because they appear embarrassed. Thereby, the important differences between positive and negative emotion in our study could be related to social rules already integrated in young children.

Based on previous studies, we expected that girls would produce positive FE with better quality than boys, and that boys would produce negative FE with better quality than girls (LaFrance et al., 2003; Komatsu and Hakoda, 2012; Chaplin and Aldao, 2013). We did find a significant interaction between gender and anger FE. Boys are better for producing anger than girls. Girls did not significantly produced joy with more quality than boys. However, we also found a significant interaction between age, gender and emotion subtype for joy, sadness, and neutral meaning that the differences between boys and girls may change according to age. Our results join the results of Chaplin and Aldao (2013) who also found a significant interaction between age, gender and emotion. We also looked at the effect of avatars gender on the productions of FE but found no significant effect. Boys and girls produced FE in a similar way, whatever the gender of the avatar. However, the quality of the children’s production may depend of the quality of avatars. The fact that these avatars were previously rated by adults rather than children may bias the validity of the stimuli material when used on children.

We also expected that children would be helped by the bimodality. However, we found no effect of the modality on the productions of FE. Specifically, the presence of sound did not support the children’s productions. In the bimodality, it appears that sometimes children can produce a correct sound the FE does not concur with the emotion targeted. In these cases, the annotator tends to pay more attention to the FE than the sound for two reasons: (i) FE are social signals that convey more strongly the information of the emotion felt than sound, (ii) the dataset was created to design an algorithm for automated facial recognition to be integrated in a serious game for ASD (Grossard et al., 2017). As a consequence, it is possible that raters considered that the most important information to rate was the facial signal. This tendency to pay more attention to FE than sound could modulate the effect of the modality.

We also expected an effect of the task on the children’s productions. We proposed two different tasks, (i) one task of production with a model, the imitation task, (ii) one task of production without model, the on request task. We expected that children would perform better in imitation task because the model could help children in their productions. However, children significantly produced FE of better quality in the on request task than in imitation task. In fact, during the imitation task, children tried to stick as well as possible to the model. They did not need to understand the played emotion and tended to just analyze the placement of the elements on the avatar’s face. Indeed, the productions were not always credible but also sometimes not well recognizable. In contrast, in the on request task, children had to themselves represent what the emotion triggers in order to produce the correct FE. This conscious control due to representation of the emotion requested to the child may be reparable because for somehow, they have a more important latency before starting their productions (subjective impression of raters but not objectively measured). Thereby, their productions tended to be closer to a real spontaneous expression, and also more credible.

The worse results in the imitation task could also come from our choice to use avatars instead of real persons to support the productions of the children. We choose avatars because of the interest of people with ASD for virtual environment (Boucenna et al., 2014). In a future work, we will propose our protocol to children with ASD and will compare their results to the results of typical developing children.

We also studied the effect of the site on the productions of the children’s FEs. We found a significant effect between the two locations, in favor of children from Nice. This effect is subtle, as the size effect is not large. There are two ways to interpret this result. (i) The site effect is likely due to cultural factors as people in the south of France and the Mediterranean coast in general tend to be known as more expressive than those from Parisian. These findings concur with the literature that reports an effect of social environment on the production of FE (Camras et al., 2006). (ii) As the annotators were Caucasian and there were more Caucasian children recruited in Nice (89.7%) than in Paris (58.7%), judges might have been more accurate in recognizing FE on Caucasian children. These observations concur with the in-group advantage in emotion recognition (Elfenbein and Ambady, 2002).

Finally, the way to rate the productions of typical children was adapted to the requirements of the game as well as the design of the algorithm that will be implemented in the serious game. The choice of rating the credibility and the use of four scales at a time may have influenced the ratings. However, we obtained an excellent agreement between judges who rated the videos and our results are in accordance with the literature. Moreover, our coding procedure mixed recognition and credibility. Thinking of neutral emotion, what a credible neutral expression is may be odd to understand (e.g., no movement, only opening mouth). Since we are working on an algorithm that should recognize emotional and neutral FE we had to keep the same scoring for all FE. However, this limitation is more theoretical than empirical, since we had very few ambiguous neutral FE (10% scores between 3 and 7) in the dataset.

## Conclusion

In this study, we evaluated the effect of different moderators on the productions of FEs in children between 6 and 11 years old. We found that age, emotion, task and cultural environment modulate their productions. Also, production on request was easier than production imitating an avatar model. Taking into account these variables is necessary for the evaluation of competences of typical children but also comparison with a pathological population. In a future research, we plan to propose this protocol to children with ASD in order to characterize and compare their productions to those of typical children. We will also use the dataset to train classification algorithms for FE recognition in order to integrate it into the serious game JEMImE.

## Author Contributions

CG, SH, JB, AD, and HD: conception, acquisition, and interpretation of data, drafting the work. LaC, SS, PF, MC, LiC, KB, OG, and DC: conception, interpretation of data and revising the work. HP: analysis and interpretation of data, drafting the work.

## Conflict of Interest Statement

PF is general director of Groupe Genious Healthcare, a private company that develops serious games for health purposes. The other authors declare that the research was conducted in the absence of any commercial or financial relationships that could be construed as a potential conflict of interest.

## Footnotes

1 http://www.education.gouv.fr/cid187/l-education-prioritaire.html
